# The α7 nicotinic acetylcholine receptor agonist, GTS-21, attenuates hyperoxia-induced acute inflammatory lung injury by alleviating the accumulation of HMGB1 in the airways and the circulation

**DOI:** 10.1186/s10020-020-00177-z

**Published:** 2020-06-29

**Authors:** Ravikumar A. Sitapara, Alex G. Gauthier, Sergio I. Valdés-Ferrer, Mosi Lin, Vivek Patel, Mao Wang, Ashley T. Martino, Jeanette C. Perron, Charles R. Ashby, Kevin J. Tracey, Valentin A. Pavlov, Lin L. Mantell

**Affiliations:** 1 Department of Pharmaceutical Sciences, St, College of Pharmacy and Health Sciences, St. John’s University College of Pharmacy and Health Sciences, St. Albert Hall, 8000 Utopia Parkway, Queens, New York, 11439 USA; 2 Feinstein Institutes for Medical Research, Northwell Health System, 350 Community Drive, Manhasset, New York, 11030 USA

**Keywords:** Hyperoxia, Lung injury, a7nAChR, Vagus nerve, Cholinergic anti-inflammatory pathway, Inflammatory reflex, Sterile inflammation

## Abstract

**Background:**

Oxygen therapy, using supraphysiological concentrations of oxygen (hyperoxia), is routinely administered to patients who require respiratory support including mechanical ventilation (MV). However, prolonged exposure to hyperoxia results in acute lung injury (ALI) and accumulation of high mobility group box 1 (HMGB1) in the airways. We previously showed that airway HMGB1 mediates hyperoxia-induced lung injury in a mouse model of ALI. Cholinergic signaling through the α7 nicotinic acetylcholine receptor (α7nAChR) attenuates several inflammatory conditions. The aim of this study was to determine whether 3–(2,4 dimethoxy-benzylidene)-anabaseine dihydrochloride, GTS-21, an α7nAChR partial agonist, inhibits hyperoxia-induced HMGB1 accumulation in the airways and circulation, and consequently attenuates inflammatory lung injury.

**Methods:**

Mice were exposed to hyperoxia (≥99% O_2_) for 3 days and treated concurrently with GTS-21 (0.04, 0.4 and 4 mg/kg, i.p.) or the control vehicle, saline.

**Results:**

The systemic administration of GTS-21 (4 mg/kg) significantly decreased levels of HMGB1 in the airways and the serum. Moreover, GTS-21 (4 mg/kg) significantly reduced hyperoxia-induced acute inflammatory lung injury, as indicated by the decreased total protein content in the airways, reduced infiltration of inflammatory monocytes/macrophages and neutrophils into the lung tissue and airways, and improved lung injury histopathology.

**Conclusions:**

Our results indicate that GTS-21 can attenuate hyperoxia-induced ALI by inhibiting extracellular HMGB1-mediated inflammatory responses. This suggests that the α7nAChR represents a potential pharmacological target for the treatment regimen of oxidative inflammatory lung injury in patients receiving oxygen therapy.

## Introduction

Patients with hypoxemia and acute respiratory failure frequently require the administration of hyperoxia (inspired content of O_2_ > 21%) (Carvalho 1998; Snider and Rinaldo 1980; Han and Mallampalli 2015). In this context, hyperoxia can become a lifesaving intervention in clinical scenarios as diverse as preterm newborns, adults with respiratory distress, or patients suffering from severe acute respiratory decline, such as patients with COVID-19 (Windhorst et al. 2009; Silversides and Ferguson 2013; Giwa et al. 2020; Guan et al. 2020; Huang et al. 2020). However, prolonged exposure to hyperoxia is associated with oxygen toxicity, due to the excessive production of reactive oxygen species (ROS), which can lead to oxidative stress-mediated acute inflammatory lung injury, known as hyperoxia-induced acute lung injury (HALI) (Kallet and Matthay 2013; Morrow et al. 2007; Matthay and Zimmerman 2005).

This oxidative stress-mediated HALI is characterized by 1) an influx of inflammatory cells, including polymorphonuclear neutrophils (PMNs); 2) enhanced cytokine production; 3) injury and death of pulmonary epithelial and endothelial cells; 4) pulmonary proteinaceous edema; 5) destruction of the alveolar-capillary barrier; and 6) impaired gas exchange, leading to the severe morbidity and mortality in patients on ventilation (Bhandari and Elias 2006; Crapo 1986; Slutsky 1999; van Zoelen et al. 2008). In this context, pro-inflammatory chemokines and cytokines have been implicated in neutrophil recruitment into the lungs (Jiang et al. 2017; Matthay et al. 2012; Belperio et al. 2002; Olson and Ley 2002). However, the specific molecular mechanisms underlying cytokine-mediated HALI remain to be elucidated. Clinically, there are no efficacious treatments that significantly reduce the inflammatory lung injury in patients receiving oxygen therapy.

Previously, we have shown that prolonged exposure to hyperoxia induces the accumulation of high mobility group box-1 protein (HMGB1) in mouse airways (Entezari et al. 2014). The mechanistic role of HMGB1 in oxidative stress-induced ALI is incompletely understood; extracellular HMGB1, either actively secreted by immune cells or passively released by necrotic cells, has been implicated in the pathophysiology of a variety of inflammatory conditions (Scaffidi et al. 2002; Yang et al. 2001), such as sepsis and rheumatoid arthritis (Andersson et al. 2000; Taniguchi et al. 2003; Wang et al. 1999; Wang et al. 2004). The inactivation of HMGB1 with specific anti-HMGB1 antibodies increased the survival of animals with severe polymicrobial sepsis, reduced high tidal volume ventilation-induced lung injury, and diminished endotoxin and hemorrhage-induced increases in pulmonary levels of inflammatory cytokines (Abraham et al. 2000; Lutz and Stetkiewicz 2004; Ogawa et al. 2006; Ueno et al. 2004). In addition, an increase in the levels of HMGB1 in the plasma and lung epithelial-lining fluids has been reported in patients with ALI (Kolliputi et al. 2010; Misharin et al. 2013). Extracellular HMGB1, a potent pro-inflammatory mediator (Wang et al. 1999), can initiate an inflammatory response, facilitating the progression of sepsis and ALI (Entezari et al. 2014; Abraham et al. 2000; Kolliputi et al. 2010; Crapo et al. 1982; Liu et al. 2008). Currently, the role of HMGB1 in oxidative stress-induced ALI remains to be elucidated. We have previously shown a significant correlation between airway HMGB1 levels and oxidative lung injury induced by hyperoxia (Entezari et al. 2014). The exposure of mice to hyperoxia (≥99% O_2_) results in an increased accumulation of HMGB1 in the mouse airways and the administration of anti-HMGB1 antibodies significantly reduced the severity of HALI (Entezari et al. 2014). These results indicate that the high levels of airway HMGB1 play a critical role in lung injury induced by oxidative stress under hyperoxic conditions. Therefore, targeting the accumulation of airway HMGB1 may be of benefit in the treatments of patients with HALI.

The activation of α7 nicotinic acetylcholine receptors (α7nAChRs) plays a critical role in mediating the vagus nerve-based inflammatory reflex and specifically its efferent arm, termed the *cholinergic anti-inflammatory pathway* (Wang et al. 2003; Borovikova et al. 2000). Its activation results in decreased translocation of HMGB1 from the nucleus to the cytoplasm, with subsequent release into the extracellular milieu (Wang et al. 2003; Brewer et al. 1996; Bustin 2001; de Jonge and Ulloa 2007; Pavlov et al. 2007). Lung cells express high levels of α7nAChR, which could be targeted to reduce the accumulation of extracellular HMGB1 (Bustin 2001; Pavlov et al. 2007; Calogero et al. 1999). GTS-21, a partial agonist of α7nAChR (Chastre and Fagon 2002; Cook et al. 1998), has been previously used therapeutically for experimental sepsis and in other preclinical conditions (Pavlov et al. 2007; Kang et al. 2014; Tarnawski et al. 2018; Mavropoulos et al. 2017). GTS-21 has been used in human studies and has a favorable safety profile at doses up to 450 mg/day (Kitagawa et al. 2003; Kox et al. 2011). The aims of this study were to determine the effects of GTS-21 on (a) the accumulation of extracellular HMGB1 in the animals subjected to prolonged exposure to hyperoxia, (b) attenuating hyperoxia-induced lung injury and (c) hyperoxia-induced pulmonary pro-inflammatory responses, especially the infiltration of inflammatory leukocytes into the airways and lung tissues.

## Materials and methods

### Special reagents

GTS-21 was obtained from Abcam (Cambridge, MA, USA). The pH of the GTS-21 solution was adjusted to 7 before intra-peritoneal injection in mice. 0.9% normal saline solution was used as the control vehicle.

### Animal studies

C57BL/6 mice (male, 8 to 12 weeks old; The Jackson Laboratory, Bar Harbor, ME, USA) were used in this study, in accordance with the Institutional Animal Care and Use Committees of St. John’s University. The mice were housed in a specific pathogen-free environment maintained at 22 °C in ≈50% relative humidity with a 12-h light/dark cycle. All mice had ad libitum access to standard rodent food and water. Mice were exposed to hyperoxia as previously described (Patel et al. 2013). Briefly, animals were placed in micro isolator cages (Allentown Caging Equipment, Allentown, NJ, USA) that were kept in a Plexiglas chamber (BioSpherix, Lacona, NY, USA) and exposed to ≥99% O_2_ or remained in room air for 72 h. Mice exposed to hyperoxia were randomized to receive either intraperitoneally administered GTS-21 (0.04, 0.4 and 4 mg/kg) or the control vehicle, saline, every 8 h, starting 32 h after the onset of hyperoxic exposure. At the end of hyperoxic exposure, mice were euthanized with intraperitoneal sodium pentobarbital (120 mg/kg) to obtain bronchoalveolar lavage (BAL) fluid samples or lung tissues, as described below, for further analysis.

### Bronchoalveolar lavage fluids and serum collection

Murine BAL fluid containing proteinaceous debris and cells was obtained as previously described (Patel et al. 2013; Sitapara et al. 2014). Briefly, mice were euthanized by an intraperitoneal injection of sodium pentobarbital (120 mg/kg). Following a 1- to 2-cm incision made on the neck, the trachea was dissected and a 20-gauge × 1.25-in. intravenous catheter was inserted caudally into the lumen of the exposed trachea. The lungs were gently lavaged twice with 1 mL of a sterile, nonpyrogenic phosphate-buffered saline (PBS) solution (Mediatech, Herndon, VA, USA). The BAL samples were centrifuged at 4 °C at 3200 x g and the resultant supernatants were stored at − 80 °C and pellets used for cell content analysis. For serum collection, whole blood was collected by cardiac puncture and allowed to coagulate for 30 min at room temperature. Next, samples were centrifuged at 2000 x g for 10 min at 4 °C and the resulting supernatant was stored as serum samples. Proteins in normalized volumes of BAL and serum samples were separated by SDS-PAGE and HMGB1 concentrations were determined by Western blot analysis as described below. Total BAL protein content was analyzed by bicinchoninic acid (BCA) assay in BAL samples. For cell content analysis in BAL samples, cell pellets were subjected to red blood cell lysis. Next, differential cell analysis was performed by depositing a monolayer of cells onto a cover slip using a Shandon Cytospin 2 centrifuge (Marshall Scientific, Hampton, NH) and then stained with Hema 3 solution kit (Fisher Scientific, Hampton, NH) per the manufacturer’s instructions or by flow cytometry analysis as described below.

### Measurement of HMGB1

The concentrations of HMGB1 in BAL samples were determined using immunoblotting analysis with an anti-HMGB1 antibody, as previously described (Wang et al. 1999). In brief, samples were separated first on SDS-PAGE. Next, the proteins were electrotransferred to a polyvinyledene difluoride membrane, and blocked with 5% nonfat dry milk in Tris-buffered saline with 0.1% Tween-20 (TBST). The membrane was incubated with anti-HMGB1 (1:1000), washed with TBST, followed by incubation with a goat anti-rabbit horseradish peroxidase-coupled secondary antibody (1:10,000) (BioRad, Hercules, CA). After washing, antibody binding was detected using Enhanced Chemiluminescence Plus Western blotting detection reagents (Amersham Pharmacia Biotech, Piscataway, NJ). Western blots were scanned with a UVP Biospectrum 600 Imaging System (Vision Works LS, Upland, CA), and the band intensities were quantified using Image J analysis software version 2.0.0 (Works LS, Upland, CA).

### Histopathology

Histopathological evaluation was conducted in paraffin-embedded tissues as previously described (Entezari et al. 2014). Prior to removal from the animal, the lungs were instilled with buffered formalin solution through a 20-gauge angiocatheter placed in the trachea. The lungs were then immersed in buffered formalin overnight and processed for conventional paraffin histology. The sections were stained with hematoxylin and eosin and examined with an Evos XL core microscope (Life Technologies, Grand Island, NY). Lung injury scores were performed by three blinded independent investigators using the scoring criteria described in Szarka et al. 1997.

### Immunohistochemistry

Macrophage/monocyte staining was performed on cryosectioned lung tissue as previously described (Martino et al. 2011). Briefly, before removal from the animal, the lungs were rinsed with PBS and then instilled with 30% sucrose solution through a 20-gauge angiocatheter placed in the trachea. The lungs were frozen in an optimal cutting temperature (OCT) solution. The sections from lung tissue blocks cryopreserved in OCT were mounted on Superfrost plus slides. The sections were fixed for 10 min at room temperature (RT) in acetone and then air-dried for 15 min. An HRP immunohistochemistry staining protocol for CD11b was used with hematoxylin counter stain and mounted with Vectamount AQ aqueous mounting media (Vector Labs, Burlingame, CA). In brief, the sections were blocked (5% goat serum in PBS) for 30 min at RT, followed by PBS rinse. Biotinylated CD11b primary antibody (Thermo Scientific, Waltham, MA) was added at a 1:100 dilution in PBS with 1% goat serum for 60 min at RT, or with anti-HMGB1 antibody (Cell Signaling Technologies, Danvers, MA), at a 1:100 dilution overnight at 4 °C, followed by a PBS rinse. Peroxidase was blocked for 10 min at RT with peroxide suppressor (Thermo Scientific). After the PBS rinse, streptavidin-HRP (BD Biosciences, San Jose, CA) was added at a 1:100 diluted in PBS with 1% goat serum for 30 min at RT in the dark. Next, slides were stained with a 3,3- diaminobenzidine (DAB) buffer kit and hematoxylin stain (Vector Labs) per the manufacturer’s protocol. At least 15 images per slide were captured at 200x magnification using an inverted light microscope. CD11b positive cells were counted manually from captured images. Data were calculated per square centimeter.

### Flow Cytometric analysis

BAL cells were incubated for 10 min at 4 °C with an anti-mouse CD16/32 antibody (1:200; Biolegend, San Diego, CA) to block non-specific binding, followed by incubation with a FITC-conjugated anti-mouse CD11b (1:200; Biolegend, San Diego, CA) and an AF 700-conjugated anti-mouse CD11c (1:200, Biolegend) antibodies for 30 min at 4 °C. Data were collected on a BD LSR Fortessa flow cytometer using FACSDiva software (Becton Dickinson, Mountain View, CA) and analyzed using FlowJo software (Tree Star, San Carlos, CA). A range of 20,000–50,000 cells was analyzed per sample. The initial gating eliminated debris and red blood cells. Next, consecutive gates, were set to identify CD11c^+^ and CD11b^+^ cells, as previously described (Swirski et al. 2009; Valdés-Ferrer et al. 2013). The relative expression of CD11c was used to distinguish CD11c-positive alveolar macrophages (CD11c^+^, CD11b^+^) and CD11c-negative infiltrated cells (CD11c^neg^, CD11b^+^). Fluorescence minus one (FMO) controls were used for proper gating.

### Statistical analysis

All the data was analyzed using GraphPad Prism statistical software. The results are presented as the mean ± SEM. The data were analyzed for statistical significance according to unpaired t-test, and analysis of variance (ANOVA) with Dunnett’s post hoc analysis. The survival data were analyzed using the Kaplan-Meier analysis. The a priori *p* value was *p* < 0.05.

## Results and discussion

**Fig. 1:**
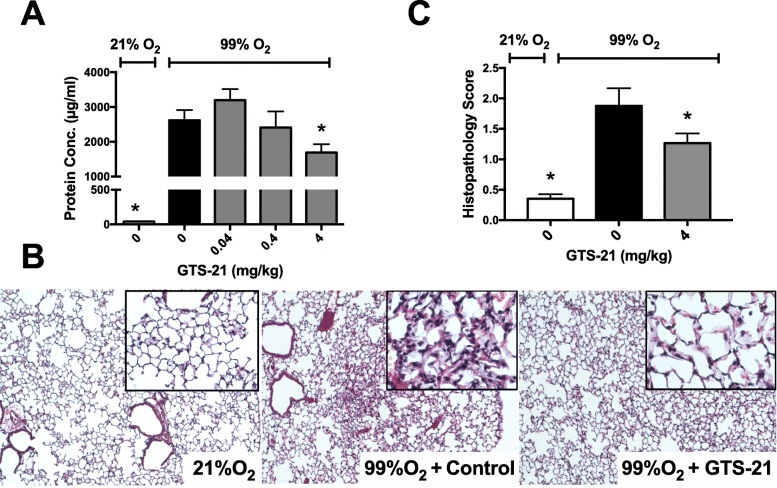
Systemic administration of GTS-21 decreases lung injury in mice exposed to prolonged hyperoxia. C57BL/6 mice were exposed to either ≥99% O_2_ for 3 days or 21% O_2_ (room air). Mice were randomized to receive either GTS-21 (0.04, 0.4 and 4 mg/kg) or saline, administrated by intraperitoneal injection every 8 h starting at 32 h following the onset of hyperoxic exposure. Lungs and BAL were harvested at the end of hyperoxia treatment. **a** The total protein content in the BAL was measured as a marker of lung injury. **b-c** Lungs, harvested at the end of hyperoxic exposure, were fixed, embedded and sectioned. Images of the Hematoxylin-eosin-stain of the lung sections from room air samples (RA), and hyperoxic samples from mice treated with either vehicle saline control (0 m/kg) or GTS-21, (4 mg/kg) are shown (**b**) and their lung histopathological scores assessed (**c**). Data represent the mean ± SEM from three independent experiments (*n* = 6–10 mice/group). *p < 0.05, compared with mice receiving normal saline

Prolonged exposure to hyperoxia induces the accumulation of HMGB1 in the airways of the exposed subjects, which can be a critical contributor to HALI (Entezari et al. 2014; Abraham et al. 2000; Ueno et al. 2004; Wang et al. 2019). Similar to other types of acute lung injury, HALI is characterized by the injury/damage of alveolar epithelial cells and pulmonary endothelial cells. The resulting impairment of the alveolar barrier can be assessed by measuring the protein content released into the airways and the presence of elevated leukocyte counts (Doerschuk 2000; Barnett and Ware 2011; Mokra and Kosutova 2015; Pittet et al. 1997). As shown in Fig. 1a, hyperoxia-induced inflammatory lung injury was characterized by increased protein leakage into the airways (Entezari et al. 2014), and 4 mg/kg i.p. of GTS-21 partially rescued mice from hyperoxia-induced pulmonary accumulation of proteins in the airways (1687 μg ± 242 μg of protein/mL, versus 99% O_2_ control vehicle 2618 ± 298 μg of protein/mL, *p* < 0.05). Similarly, 4 mg/kg i.p. of GTS-21 significantly attenuated hyperoxia-induced inflammatory lung injury (Fig. 1b and c). As we have previously reported, there is a significant positive correlation between the hyperoxia-induced inflammatory lung injury, including the infiltration of neutrophils, and the severity of HALI in mice exposed to hyperoxia (Entezari et al. 2014). This damage is also observed in mice exposed to hyperoxia and subjected to control vehicle treatment, as compared to mice that remained in room air (Fig. 1b and c, 1.88 ± 0.29 versus 0.352 ± 0.07 of the room air control, *p* < 0.05). Hyperoxia-induced acute inflammatory lung injury was attenuated by the administration of 4 mg/kg i.p. of GTS-21 (Fig. 1b and c, 1.26 ± 0.16 score versus control vehicle 1.88 ± 0.29 score, *p* < 0.05). Hyperoxia induces excessive production of ROS, causing oxidative stress-mediated ALI (Bhandari and Elias 2006; Zhang et al. 2003). The increased infiltration of ROS-releasing neutrophils can result in further oxidative stress-induced cell damage and lung injury (Steer et al. 2013; Han et al. 2018). Oxidative stress has also been implicated in LPS-induced ALI, which produces an increase in neutrophil recruitment to the lung, pulmonary cell apoptosis and an increase in the airway levels of HMGB1, TNFα and polymorphonuclear cells (Xie et al. 2012).

**Fig. 2:**
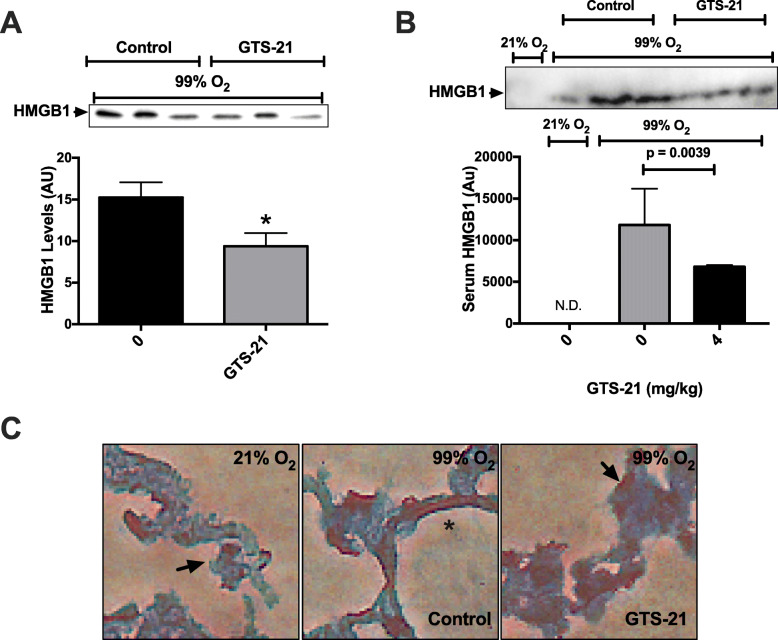
GTS-21 inhibits hyperoxia-induced translocation and accumulation of HMGB1 in the mouse airways and circulation. C57BL/6 mice were exposed to either 21% O_2_ (room air) or to ≥99% O_2_ and treated with vehicle (0 mg/kg) or GTS-21 (4 mg/kg), as described in Fig. 1. Bronchoalveolar lavage, whole blood, and lungs were harvested at the end of hyperoxia exposure. **a** A representative image of Western blots showing immunoreactive bands of HMGB1 in the mouse BAL samples. The bar graph shows the integrated density value of the HMGB1 bands of the BAL of hyperoxic mice, administered with normal saline or GTS-21 (*n* = 10 mice/group). **b** A representative Western blot for HMGB1 in mouse serum and the integrated density values for each group (N.D., not detected) (*n* = 3 mice/group). **c** Representative immunohistochemical staining for HMGB1 in mouse lungs (arrows indicate HMGB1 in nucleus, asterisks represent HMGB1 present in cytoplasm) (*n* = 3 mice/group). *p < 0.05, compared with mice receiving normal saline

Extracellular HMGB1, either actively secreted by immune cells or passively released by necrotic cells, has been implicated in the pathophysiology of a variety of inflammatory diseases (Scaffidi et al. 2002; Yang et al. 2001; Bonaldi et al. 2003). Airway HMGB1 induces significant pro-inflammatory responses in the lungs of mice exposed to hyperoxia (Entezari et al. 2014). The inactivation of HMGB1 with specific, neutralizing anti-HMGB1 antibodies significantly decreases inflammatory lung injury and increases the survival of animals in several mouse models of ALI (Entezari et al. 2014; Abraham et al. 2000; Wang et al. 2019). The inactivation of HMGB1 with a neutralizing antibody or the activation of α7nAChR with acetylcholine or GTS-21 inhibits the receptor for advanced glycation end products (RAGE)-mediated endocytosis of extracellular HMGB1 and HMGB1-LPS complexes, resulting in in a decrease in TNFα secretion and cell death in macrophages (Yang et al. 2019). ALI (induced by either polymicrobial sepsis, high tidal volume ventilation, endotoxin, or hemorrhage) is attenuated by the administration of anti-HMGB1 antibodies, which may be a result of decreased pro-inflammatory responses (Abraham et al. 2000; Lutz and Stetkiewicz 2004; Ogawa et al. 2006; Ueno et al. 2004). Accordingly, this study evaluated the levels of extracellular HMGB1 in both BAL (Fig. 2a) and serum samples (Fig. 2b), obtained from animals exposed to either hyperoxia or remained in room air. Similar to previous observations (Entezari et al. 2014), mice exposed to hyperoxia have significant levels of airway HMGB1 (Fig. 2a) (Entezari et al. 2014). The administration of 4 mg/kg i.p. of GTS-21 significantly reduced HMGB1 concentration in the airways (Fig. 2a) (9.38 ± 1.57 A.U. versus control vehicle 15.24 ± 1.8 A.U., *p* < 0.05). Next, we determined whether HMGB1 perturbation in the lung appears only in the airways. As shown in Fig. 2b, no detectable HMGB1 was observed in the circulation, reflecting our previous findings (Entezari et al. 2014). While exposure to hyperoxia for 3 days caused a markedly accumulation of extracellular HMGB1 in the serum, GTS-21 (4 mg/kg) significantly attenuated this accumulation (0.6 × 10^4^ ± 0.02 × 10^4^ A.U. versus control vehicle 1.2 × 10^4^ ± 0.4 × 10^4^ A.U. *p* < 0.05). Moreover, immunohistochemical analysis indicated (Fig. 2c) that hyperoxia-induced HMGB1 nuclear-to-cytoplasmic translocation in lung structural cells, as previously reported (Entezari et al. 2014), a critical step required for HMGB1 secretion, was significantly suppressed in GTS-21 (4 mg/kg i.p.) treated mice. We and others have shown that GTS-21 1) inhibits HMGB1 release from immune cells that were stimulated by hyperoxia (Sitapara et al. 2014) or LPS and 2) decreases levels of serum HMGB1 in a mouse model of endotoxemia (Pavlov et al. 2007; Rosas-Ballina et al. 2009). Taken together, these data suggest that GTS-21 attenuates HALI by significantly reducing the accumulation of extracellular HMGB1, attenuating its inflammatory effects both systematically and in the lung and subsequently ALI in hyperoxia-exposed subjects.

**Fig. 3:**
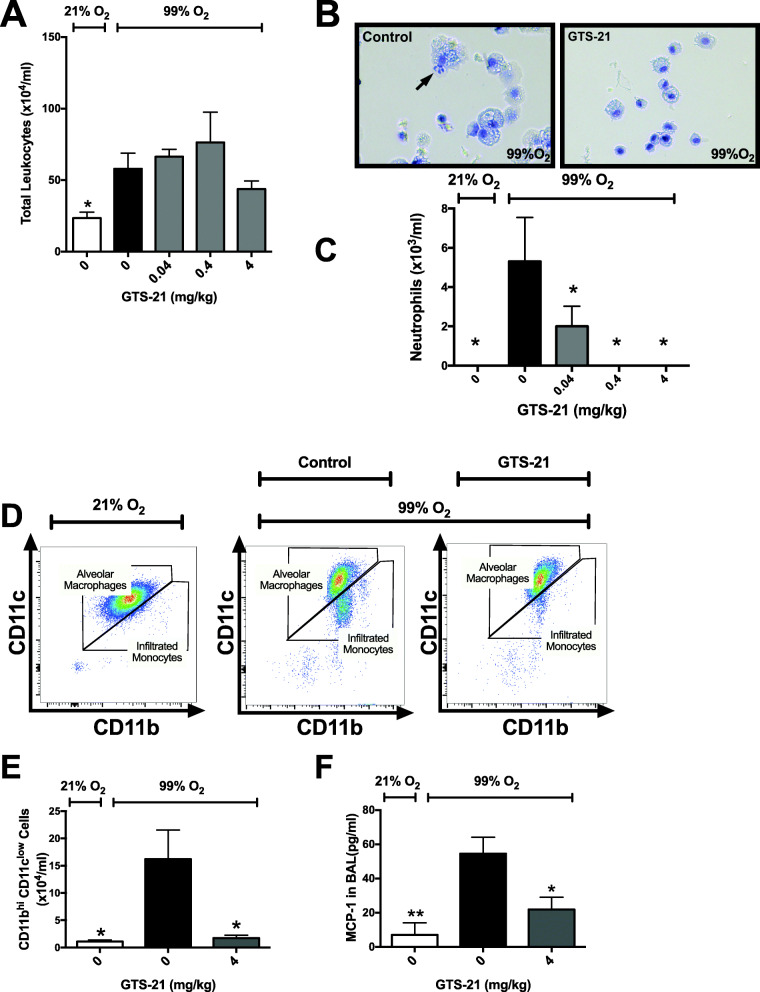
Systemic administration of GTS-21 inhibits infiltration of neutrophils and monocytes into the airways of mice exposed to hyperoxia. C57BL/6 mice were exposed to room air (21% O_2_) or hyperoxia (≥99% O_2_) and treated with vehicle (0 mg/kg) or GTS-21 (4 mg/kg), as described in Fig. 1. **a** The total amount of leukocytes in the BAL were determined by hemocytometer. **b** Cells from the BAL samples were analyzed using differential cell staining. Arrows show the presence of neutrophils stained with eosin and methylene blue in BAL samples. **c** The total number of infiltrated neutrophils in the BAL samples. **d-f** Cells from BAL were labeled with anti-CD11b and anti-CD11c antibodies and analyzed by flow cytometry (**d-e**); MCP-1 were analyzed by ELISA (**f**). **d** Representative gating for CD11b^+hi^ and CD11c^low^ cells in the BAL. **e** The graph is a representative of two independent experiments. **f** The bar graph for MCP-1 expression in BAL. Data is represented as the mean ± SEM (*n* = 2–6 per group). *p < 0.05, compared with mice receiving normal saline

Interestingly, the increase in the levels of extracellular HMGB1 in the airways and serum (Fig. 2) occurred concomitantly with a significant increase in the infiltration of leukocytes into the airways in mice subjected to prolonged exposure to hyperoxia (Fig. 3a). Similar to other inflammatory diseases, leukocyte infiltration plays a pivotal role in facilitating hyperoxia-induced inflammatory lung injury (Kang et al. 2014; Wang et al. 2019; Grommes and Soehnlein 2011). Although GTS-21 did not significantly reduce the infiltration of leukocytes into the airways, a small number of leukocytes were present in the airways of mice treated with 4 mg/kg i.p. of GTS-21 (Fig. 3a). The increased levels of airway HMGB1 have been reported to exacerbate ALI due to neutrophilic inflammation (Andersson and Tracey 2011). Extracellular HMGB1 produces inflammation due to its interaction with certain chemokine and cytokine receptors (Entezari et al. 2014; Wang et al. 1999; Abraham et al. 2000; Entezari et al. 2012). We and others have reported that the intra-tracheal administration of HMGB1 induces a significant inflammatory response characterized by the infiltration of neutrophils into the lungs of mice (Entezari et al. 2014; Abraham et al. 2000). In addition, in a mouse model of HALI, we have shown that HMGB1 accumulates in the airways prior to infiltration of neutrophils into the airways and the onset of lung injury (Entezari et al. 2014). In the present study, the attenuated extracellular HMGB1 accumulation in the airways and serum of mice treated with 4 mg/kg i.p. of GTS-21 was not accompanied by a significant reduction in total leukocyte counts in the airways (Fig. 3a). However, there was a significant dose-dependent decrease in the infiltration of neutrophils (Fig. 3b and c). Furthermore, the administration of 4 mg/kg i.p. of GTS-21 significantly decreased the infiltration of CD11c^low^ CD11b^hi^ monocytes into the airways (1.71 ± 0.52 × 10^4^ versus control vehicle 16.19 ± 5.36 × 10^4^/ml, *p* < 0.05, Fig. 3d and e). Congruent with this finding, the levels of monocyte chemotactic protein-1 (MCP-1), a proinflammatory cytokine involved in the recruitment of monocytes (Lim et al. 2015), were significantly increased in the airways of hyperoxic animals and this increase was significantly attenuated in mice treated with 4 mg/kg i.p. of GTS-21 (*p* < 0.05, Fig. 3f). Thus, these data suggest that the administration of GTS-21 attenuates HALI by decreasing the accumulation of airway HMGB1 and MCP-1, leading to a decrease in the infiltration of neutrophils and monocytes in the airways. These results are consistent with previous reports showing that α7nAChR agonists such as nicotine, PNU-282987 and DMAB-inhibit leukocyte infiltration in the lungs in mouse models of endotoxemia and *E. coli* pneumonia (Pavlov et al. 2007; Su et al. 2010).

**Fig. 4:**
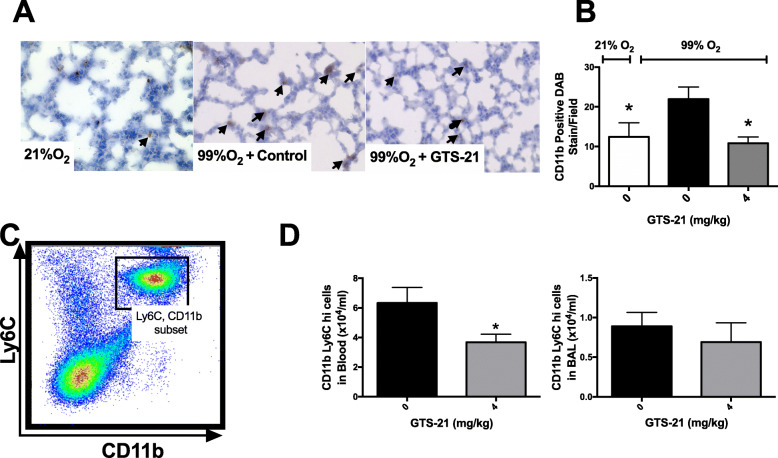
The sytemic administration of GTS-21 inhibits the infiltration of CD11b^+^ monocytes/macrophages in the lungs of mice exposed to hyperoxia. C57BL/6 mice were exposed to ≥99% O_2_ and treated with vehicle (0 mg/kg) or GTS-21 (4 mg/kg) as described in Fig. 1. The lungs were perfused with sucrose, frozen in OCT and cryosectioned. Immunohistochemistry was performed to detect CD11b. **a** Representative images of lung sections showing DAB positive cells from either control or GTS-21-treated mice. **b** The total number of CD11b positive DAB stain per field. BAL was harvested at the end of hyperoxia exposure. **c-d** Cells from the BAL and blood samples were labeled with CD11b and Ly6c antibodies and analyzed by flow cytometry. **c** Gating for CD11b^+^ and Ly6c cells in the BAL is shown. **d** The quantification of the total number of CD11b^+^ and Ly6c^+^ in BAL and blood samples analyzed by flow cytometry. Data represent the mean ± SEM from two independent experiments (*n* = 4 mice/group)

The results shown in Fig. 1b and c indicate that prolonged exposure to hyperoxia can significantly increase the lung injury score, which includes increased cellularization. To determine whether the increased lung injury score in hyperoxic mice results from an increase in the infiltration of macrophages into the lung tissue, lung tissues were immunolabelled for CD11b, a pan-macrophage marker for both residential macrophages/monocytes and those recruited (Misharin et al. 2013; Yu et al. 2016; Bronte et al. 2016). The results (Figs. 4a and b) indicated that hyperoxia significantly increased the macrophages/monocytes in lung tissues (Fig. 4a and b). Furthermore, GTS-21 significantly decreased the hyperoxia-induced CD11b^+^ macrophage/monocyte infiltration in lung tissue (21.9 ± 3.0 cells/field versus control vehicle 10.8 ± 1.5 cells/field, *p* < 0.05; Fig. 4a and b).

Decreasing the infiltration of circulating pro-inflammatory monocytes into the lung has been shown to play an important role in attenuating inflammatory lung diseases (Jiang et al. 2017; Wang et al. 2019; Zhang et al. 2018). It has been suggested that the increased expression of the Ly6C surface antigen can be used to define the inflammatory status of monocytes in the circulation and after tissue infiltration (Kratofil et al. 2017). To further assess whether the macrophages/monocytes in hyperoxic mice are pro-inflammatory, the expression of Ly6C was determined in macrophages/monocytes isolated from mouse serum and lung lavage fluids. Flow cytometric analysis of macrophages/monocytes in serum and BAL fluids from hyperoxia-exposed mice treated with 4 mg/kg i.p. of GTS-21 indicated a significant reduction in the accumulation of CD11b^+^ and Ly6C^+^ hi pro-inflammatory monocytes in the blood (3.68 ± 0.52 × 10^4^ versus control vehicle 6.33 ± 1.05 × 10^4^/ml, *p* < 0.05, Fig. 4c). However, the reduction of airway inflammatory macrophages/monocytes was not significant (0.691 ± 0.243 × 10^4^ versus control vehicle 0.891 ± 0.173 × 10^4^/ml, Fig. 4d).

**Fig. 5:**
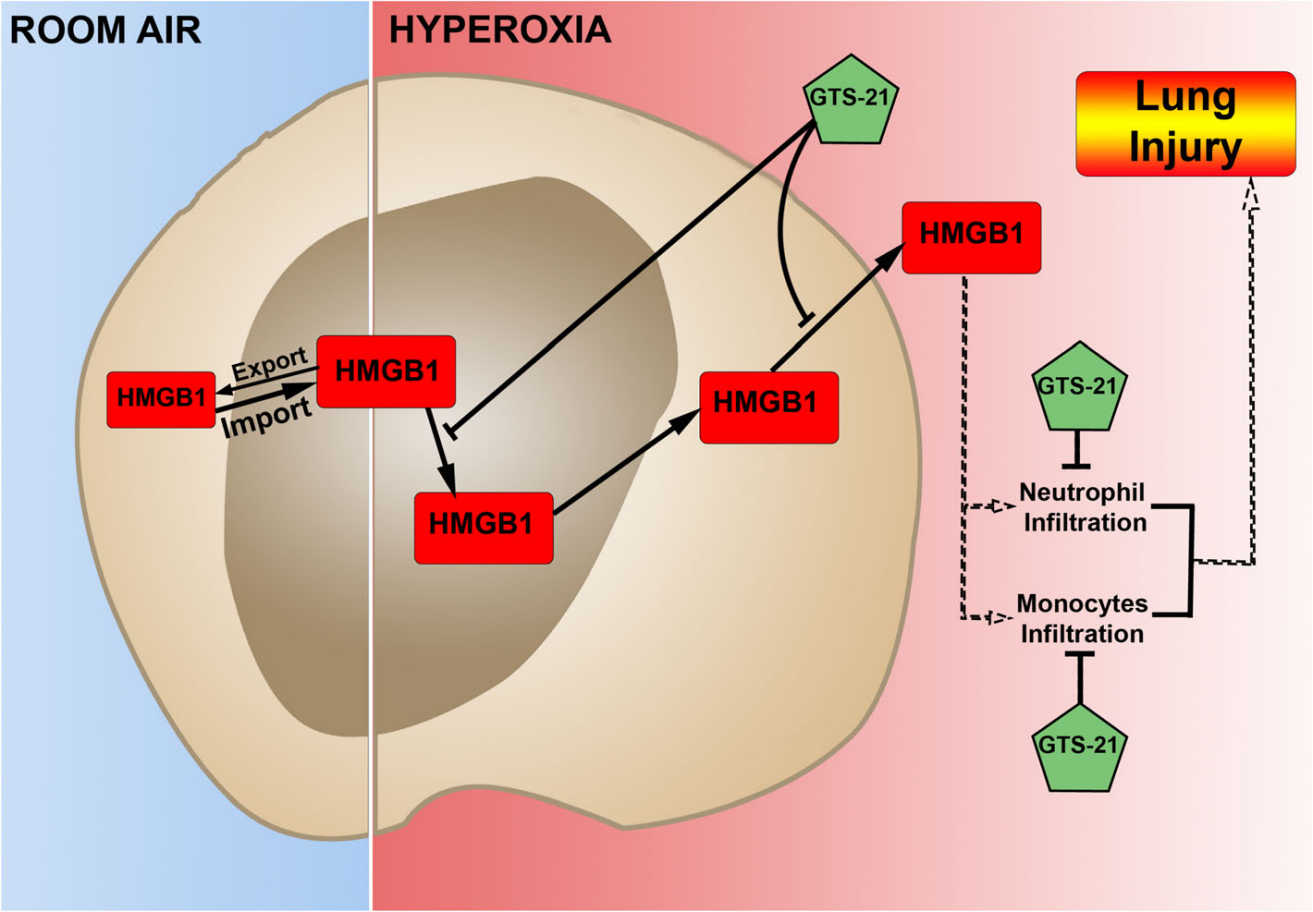
Suggested pathways of GTS-21-mediated attenuation of HALI. In cells, including lung cells, HMGB1 shuttles between the nucleus and cytoplasm. Under room air, the rate of HMGB1 nuclear import exceeds that of re-diffusion plus export. Thus, HMGB1 appears predominantly or solely in the nucleus. The prolonged exposure to hyperoxia induces HMGB1 translocation from the nucleus to the cytoplasm. The α7nAChR agonist, GTS-21, inhibits hyperoxia-induced translocation and subsequent release of nuclear HMGB1, and its accumulation in the airways and the circulation. As a result, GTS-21 is efficacious in attenuating hyperoxia-induced infiltration of leukocytes, including neutrophils and inflammatory monocytes, into the airways, thereby significantly reducing hyperoxia-induced inflammatory lung injury

The activation of α7nAChR can also inhibit endothelial cell activation, thus affecting leukocyte recruitment in acute inflammation (Saeed et al. 2005). It has been reported that the local activation of α7nAChR in the lungs by the intra-tracheal administration of either nicotine (a non-selective agonist) or PNU-282987 (a selective α7nAChR agonist) significantly attenuates the acid-induced increase in lung permeability and edema, and the protective effect of nicotine is abolished by methyllycaconitine (MLA), an antagonist of α7nAChR (Su et al. 2007). It should be noted that the protective effects of GTS-21 treatment may be due, in part, to its effect on non-α7nAChR cellular targets that produce anti-inflammatory efficacy. For example, in LPS-stimulated primary α7nAChR knockout macrophages, GTS-21 suppressed TNFα and IL-6 secretion (Garg and Loring 2019). Regardless of whether GTS-21 is functioning through α7nAChR or other receptor-mediated pathways, the results presented in this study reveal that GTS-21 has protective efficacy for HALI by attenuating several markers of inflammation (Fig. 5). Thus, this study indicates that GTS-21, an α7nAChR partial agonist, can significantly attenuate HALI by modulating the inflammatory response induced by hyperoxia.

## Conclusions

To our knowledge, this study is the first to report that the systemic administration of GTS-21 decreases hyperoxia-induced acute inflammatory lung injury. The development of HALI is due, in part, to the accumulation of extracellular HMGB1 in the airways and the circulation. GTS-21 significantly decreases the hyperoxia-induced release of HMGB1 from hyperoxia-compromised lung cells into the airways and the circulation. This, in turn, can effectively attenuate hyperoxia-induced infiltration of inflammatory neutrophils and monocytes. Therefore, targeting pathways that block the accumulation of extracellular HMGB1 by GTS-21 may provide a novel approach for developing therapies to treat oxidative stress-induced inflammatory lung injury in patients on oxygen therapy. Moreover, these findings are especially pertinent in light of the requirement for prolonged ventilation in severe cases of COVID-19.

## Data Availability

The datasets used and/or analyzed during the current study are available from the corresponding author on reasonable request.

## Abbreviations

α7nAChR: Alpha 7 nicotinic acetylcholine receptor
BAL: Bronchoalveolar lavage
GTS-21: [3-(2,4 dimethoxy- benzylidene)-anabaseine dihydrochloride]
HALI: Hyperoxia induced acute lung injury
HMGB1: High mobility group box protein 1
Hyperoxia: Greater than 99% oxygen
LPS: Lipopolysaccharide
MCP-1: Monocyte chemoattractant protein-1
PA: *Pseudomonas aeruginosa*
PMN: Polymorphonuclear cells
RA: Room air (21% Oxygen)
RAGE: Receptor for advanced glycation end-product
TNF: Tumor necrosis factor
